# Variable angle locking mesh plate for fixation of comminuted greater tuberosity fractures

**DOI:** 10.1016/j.xrrt.2025.03.006

**Published:** 2025-04-10

**Authors:** Michael E. Hachadorian, Garrett K. Berger, Karch M. Smith, William T. Kent

**Affiliations:** a Department of Orthopaedic Surgery, University of Washington, Seattle, WA, USA; b Department of Orthopaedic Surgery, University of California San Diego, San Diego, CA, USA

**Keywords:** Proximal humerus, Greater tuberosity, Fracture, Locking plate, Rotator cuff, Trauma

Isolated fractures of greater tuberosity of the humerus (AO Foundation/Orthopaedic Trauma Association 11A1.1, Fig. 1) represent a less common variant of proximal humerus fractures and, however, can still be associated with a difficulty in recovering function for some patients.^15^^,^^17^ While some of these injuries represent an avulsion with a simple fracture pattern, a large subset of these fractures is the result of impaction of the humeral head during anterior glenohumeral dislocation, resulting in a comminuted fracture fragment and impacted fracture bed.^19^ In addition to the bony injury, there is commonly an associated traumatic rotator cuff rupture which further complicates treatment.^20^

**Figure 1:**
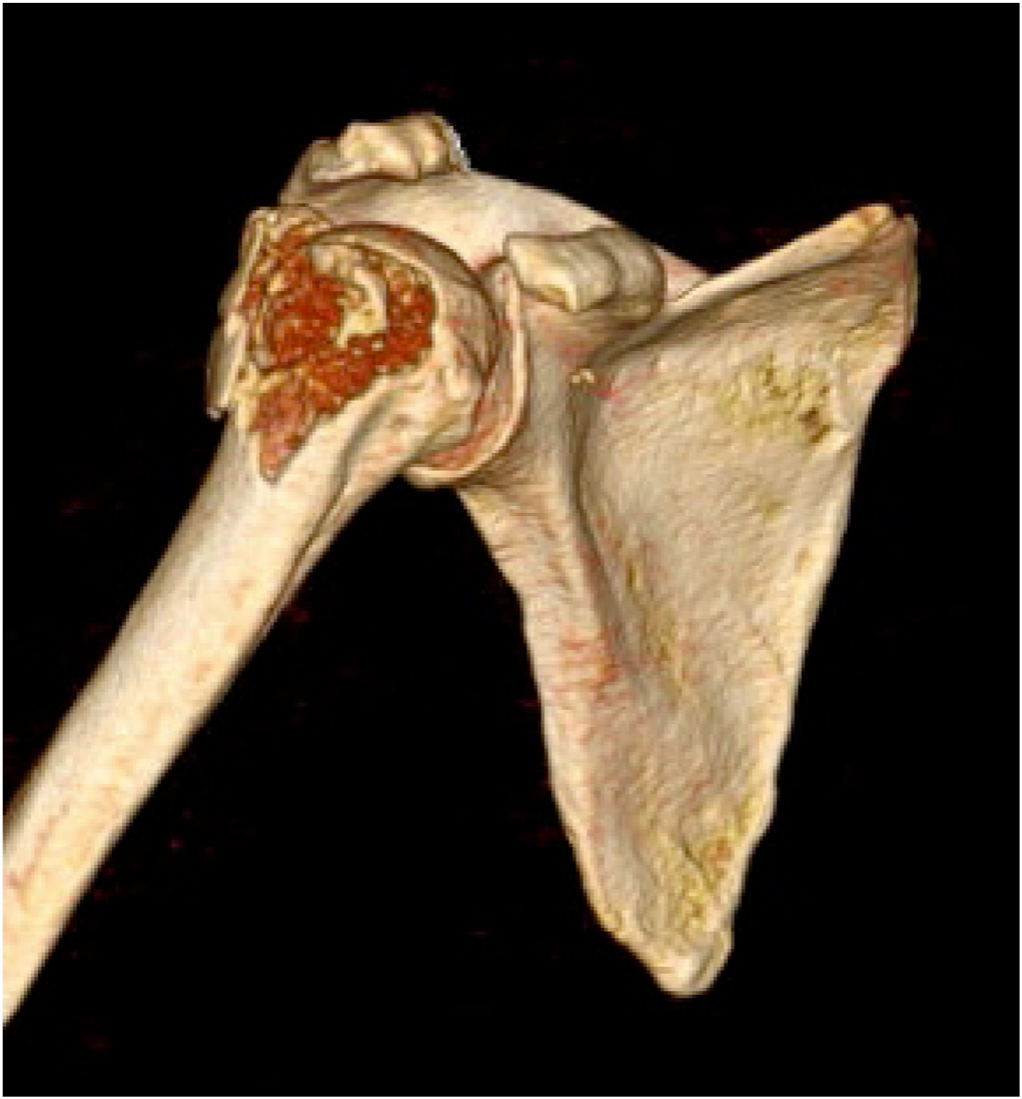
This is a CT-based 3D surface model reconstruction of a right shoulder demonstrating a comminuted fracture of the greater tuberosity that is displaced posteriorly and superiorly. Note the extension of the fracture lateral to the level of the surgical neck of the humerus. *CT*, computed tomography; *3D*, three-dimensional.

More than half of these fractures are associated with an anterior shoulder dislocation.^1^ For patients undergoing open reduction and internal fixation, up to 8% have been reported to undergo reoperation at 1 year postoperatively, with 40% of those cases being for rotator cuff repair. Furthermore, of patients who were initially managed nonoperatively, 7% went on to have surgery at 1-year postinjury, and 41% were treated with open reduction and internal fixation.^16^ With an elevated rate of subsequent operations, a thorough discussion must be had with the patient regarding the indications for surgery. However, it remains clear that both greater tuberosity fixation and rotator cuff healing are problematic in this patient population.^16^ This article describes a technique and outcomes of utilizing a combined bony and soft tissue fixation construct with the goal of reducing the risk of fracture displacement and improving patient outcomes.

## Technique

### Positioning

The patient is placed in the beach chair position with the torso elevated 30° upright. The operative shoulder and ipsilateral hip are positioned at the edge of the bed to facilitate intraoperative imaging using C-arm fluoroscopy. The operating table is rotated 90° with the contralateral extremity facing the anesthesiologist. Fluoroscopy is brought in from the patient's head. The image intensifier can “C-over” approximately 30° and be tilted towards the contralateral extremity to match the plane of the scapula to obtain a True anteroposterior (AP; Grashey) view with the arm held in slight forward flexion. If the patient's torso is elevated greater than 30°, there can be challenges obtaining this view secondary to the inability of the C-arm to “C-over” far enough. From this position, the image intensifier can “C-under” to parallel the plane of the acromion process and obtain an axillary view. Care should be taken to ensure that the image intensifier is not brought into contact with the patient's head during this maneuver. It is the author's preference to ensure that these images can be obtained prior to performing the sterile prepping and draping of the affected extremity.

### Approach/exposure

A deltopectoral approach can be utilized for exposing this fracture; however, it is the author's preference to utilize a laterally based deltoid-split approach for isolated greater tuberosity fractures. It is felt that the fracture bed can be readily exposed, and the posteriorly displaced fracture fragment can be retrieved through limited exposure. In addition, the arm can be maintained in neutral or external rotation during the process of reducing and fixating the fragment, which is felt to be advantageous to minimize tension through the attached rotator cuff musculature. A skin incision is made from the lateral border of the acromion process 4-5 cm distally. Dissection down to the deltoid fascia is carried out using electrocautery. The deltoid is split bluntly in line with its fibers until the axillary nerve is palpated distally as a band running transversely, approximately 5 cm distal to the acromion. Depending on fracture characteristics, the deltoid split can be carried distally with careful dissection and protection of the axillary nerve. The subdeltoid bursa is incised and the lateral aspect of the proximal humerus is exposed. A self-retaining retractor is used to maintain exposure. Utilization of a Kolbel or a Trimline (Medtronic, Minneapolis, MN, USA) cervical spine retractor system offers a highly adjustable, low profile self-retaining retractor with multiple blade lengths to accommodate variations in deltoid thickness. For fractures without distal extension, the smaller size of the variable angle mesh locking plate compared to a standard proximal humerus locking plate obviates the need for dissection of a window distal to the anterior branch of the axillary nerve for implant positioning. With more distal extension of the tuberosity fracture pattern, longer plate configurations with 1- or 3-hole plate extensions to the humeral metaphysis can be utilized (Video 1).

### Fracture reduction and fixation

After exposure, hematoma is débrided from the fracture site and the lateral aspect of the proximal humerus to allow for direct visualization of the reduction. A cobb is used to release adhesions in the subdeltoid and subacromial spaces. The greater tuberosity fragment is then identified and retrieved with an Allis clamp or a suture into the rotator cuff. Sutures are placed sequentially through the rotator cuff to deliver the greater tuberosity fragment into the incision. The fragment is then débrided of any hematoma or bursal tissue at the fracture site. After fragment mobilization, a series of 2 or 3 inverted mattress sutures using #2 FiberWire (Arthrex, Naples, FL, USA) are placed into the rotator cuff tendons just medial to its insertion into the greater tuberosity. The nonabsorbable sutures are used to gain a provisional reduction of the fragment, by pulling in a distal and anterior direction. It is at this time that a critical appraisal of the reduction of the tuberosity is performed. It is important to ensure that the proximal aspect of the tuberosity fragment is approximately 8 mm distal to the superior aspect of the humeral head articular surface. In comminuted fractures, volume loss resultant from an impacted fracture bed makes excessive distalization of the fragment an easy mistake to make. We prefer to provisionally fixate the fragment with multiple Kirschner wires and obtain fluoroscopic images at this point to critically evaluate the reduction.

**Figure 2:**
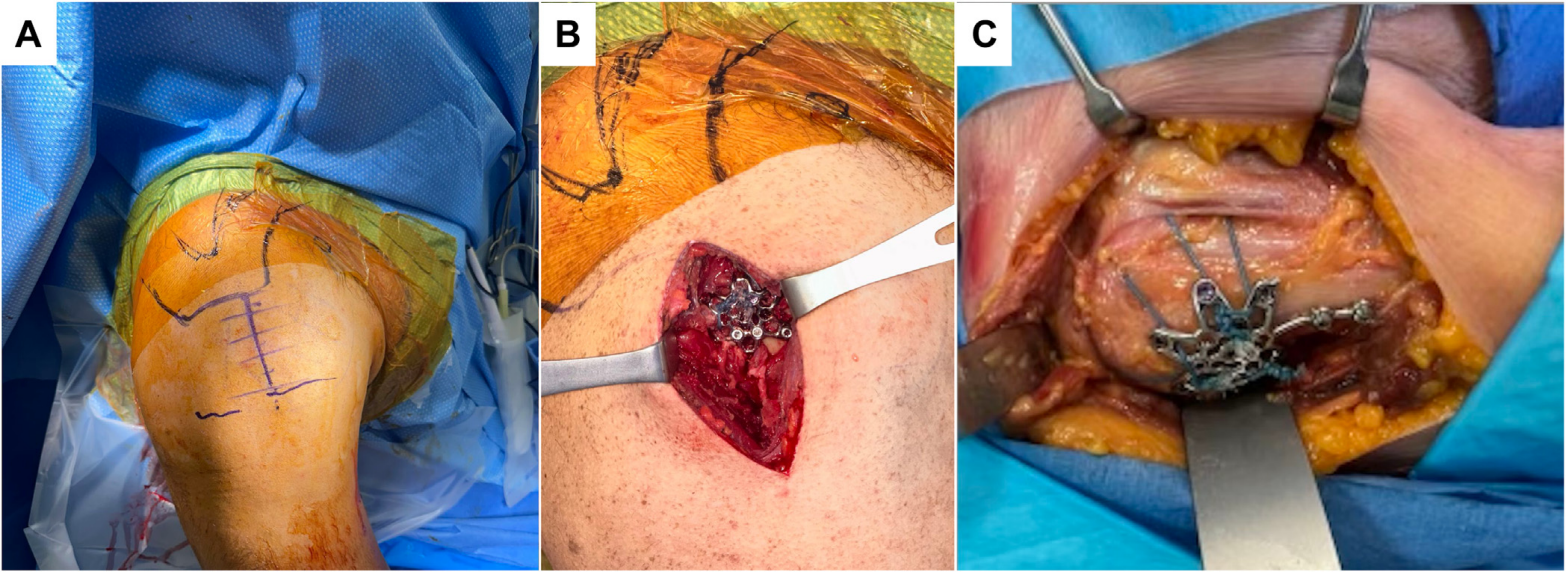
Panel (**A**) represents skin markings and positioning for the deltoid-split approach. Panel (**B**) represents an intra-operative view of the final construct and visualization attainable with this approach. Panel (**C**) is a cadaveric exposure of the same construct for perspective.

Following this, a variable-angle locking mesh plate (Depuy-Synthes, Raynham, MA, USA) is positioned over the fragment. A Freer elevator can be used to palpate the most cranial extent of the humeral head underneath the supraspinatus tendon to ensure appropriate positioning of the plate relative to this landmark. The plate is placed on the lateral aspect of the greater tuberosity. In general, the superior and posterior flanges of the plate are precontoured to wrap around the curvature of the proximal tuberosity. This is different from traditional proximal humerus plates which are generally designed to be positioned distal to the proximal margin of the greater tuberosity. In cases with significant comminution, the low-profile superior tabs of the plate can be bent to better capture the superior fragments of the tuberosity. The plate is provisionally secured to the bone using Kirschner or olive wires and the positioning of the plate is confirmed fluoroscopically using the true AP and axillary views. Ideal positioning of the plate includes overlapping the posterior fracture margin as this if felt to optimize fragment capture and minimize risk of posterior escape. Following this, cortical screws are placed centrally in the plate, to bring the plate down to the bone. Cortical screws in the plate are oriented distally to gain purchase in the better-quality bone at the medial calcar of the proximal humerus. These fractures can be fixated in a lag-by-technique fashion in the setting of simple avulsion or split fractures without significant comminution. In situ plate benders are used to contour the plate to bone after initial cortical screw fixation. Variable angle locking screws are then placed through the fragment using the variable angle locking drill guide. Screws are intentionally left short of the subchondral bone of the humeral head to avoid screw penetration into the glenohumeral joint. Variable angle locking screws are aimed away from the center of the humeral head, given that the fixed angle locking trajectories would be convergent given the plate contour. After completion of screw placement, the nonabsorbable FiberWire sutures are tensioned and tied over the plate. It is the author's preference to bring the limbs of the suture through the smooth windows in the plate avoiding threaded screw holes as this can lead to suture failure. There are multiple smooth windows available in the plate for suture passage. The window size and availability aids in the ease of suture passage through the plate. Sutures are then tied over the top of the plate creating a tension band (Fig. 2). Using a true AP and axillary fluoroscopic views of the humeral head, the humerus is brought from maximum internal rotation to maximum external rotation to ensure no screws are penetrating the medial aspect of the humeral head. The wound is irrigated thoroughly with normal saline and closed in layers with absorbable suture (Video 1).

### Postoperative protocol

Postoperatively, all patients were placed into a simple sling. Codman pendulums and active elbow range of motion (ROM) are initiated starting postoperative day 1. At 1 week postoperatively, the patient begins passive shoulder ROM with physical therapy. Active shoulder elevation is encouraged starting at 4 weeks postoperatively and strengthening to begin at 3 months postoperatively.

## Case series

**Figure 3:**
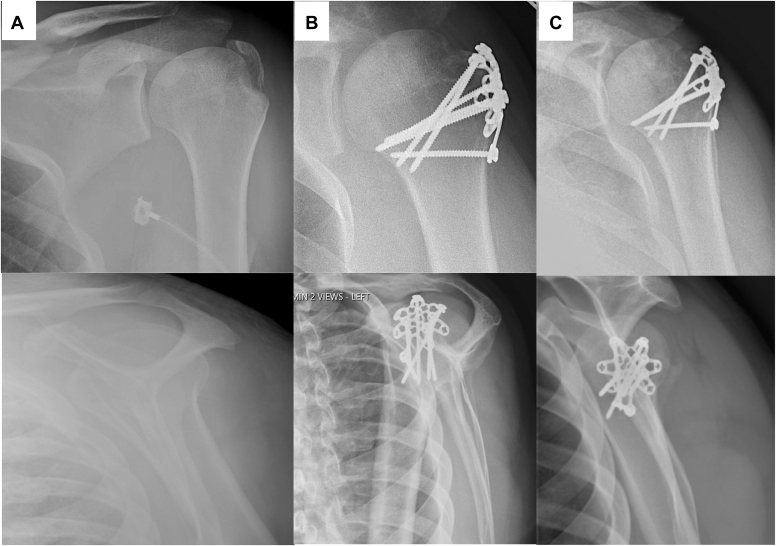
Patient 1 with anteroposterior (AP) and scapular Y views at injury (**A**), 4 months (**B**) and 1 year (**C**), displaying a healed fracture without interval displacement.

We report outcomes on four patients who underwent open reduction internal fixation of a proximal humerus fracture with a low-profile, malleable patellar plate with variable angle locking screw options supplemented by soft tissue-to-plate fixation. A single surgeon performed open reduction and internal fixation using this technique for 4 consecutive patients for isolated greater tuberosity fractures between 2021 and 2022. There were 3 males and 1 female, the average age was 41 years (range 21-74 years) and the average follow-up was 13 months (range 9-17 months). 100% of patients went on to radiographic union at an average of 4 months (Fig. 3). All patients had excellent ROM, and there were no reoperations or complications (Table I).

**Table I:** Demographic and radiographic data of four patients who underwent ORIF of the greater tuberosity of the proximal humerus.

Patient	Age	Sex	Follow-up (mos)	AFF	Abduction	ER	IR	Complications?	Union achieved?	Reoperation?
1	26	M	13	170	165	40	T11	No	Yes	No
2	22	M	11	160	90 (90)	30 (45)	T12	No	Yes	No
3	41	M	9	160	140	40	PSIS	No	Yes	No
4	75	F	17	100	--	--	--	No	Yes	No

*ORIF*, open reduction with internal fixation; *AFF*, active forward flexion; *ER*, external rotation; *IR*, internal rotation.

**Figure 4:**
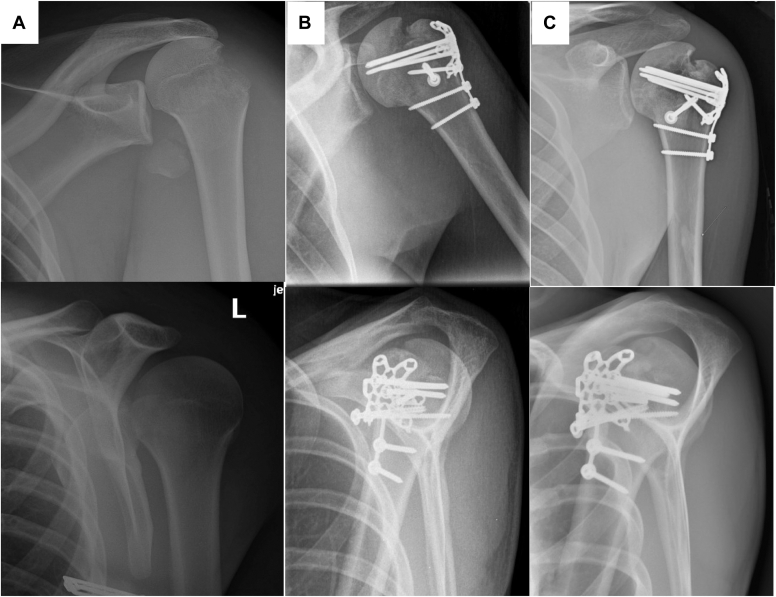
Patient 1 with anteroposterior (AP) and scapular Y views at injury (**A**), 4 months (**B**) and 1 year (**C**), displaying a healed fracture without interval displacement.

The three male patients were significantly younger than the female patient. All patients had excellent baseline function and bone quality, and as such will be discussed separately from the elderly female patient. Patient 1 is a 26-year-old male who presented as a polytrauma with bilateral femur fractures, a left clavicle fracture and a left isolated greater tuberosity fracture (Fig. 3). Following definitive management of his femur fractures, he was indicated for open reduction with internal fixation (ORIF) of his ipsilateral greater tuberosity with the described technique. Similarly, Patient 2 is a 22-year-old male who sustained an isolated right shoulder fracture dislocation (Fig. 4). Following closed reduction, he elected for ORIF of the isolated greater tuberosity fracture. Patient 3 is a 41-year-old male who sustained a left isolated comminuted greater tuberosity fracture as well as a right closed shoulder dislocation; following closed reduction of the right shoulder, he elected to proceed with ORIF of the comminuted left greater tuberosity fracture. All patients experienced uneventful postoperative courses, went on to radiographic union, and excellent ROM (Table I).

Finally, patient 4 is a 74-year-old female who had a lower baseline functional status, dementia, and poor bone quality. While treated with the same construct, her physiologic status and injury are notably different. She sustained a shoulder fracture dislocation after a mechanical ground level fall. Following closed reduction, she was noted to have an axillary nerve palsy. Once medically stable, she elected for ORIF of her isolated greater tuberosity fracture. Again, she experienced a relatively uneventful postoperative course and ultimately resides in a memory care unit, though she was noted to achieve 100° FF without pain at final follow-up.

## Discussion

Obtaining adequate fixation in comminuted and displaced fracture of the greater tuberosity can represent a challenge.^4^^,^^8^^,^^15^^,^^21^ Displacement of the greater tuberosity fragment >5 mm is historically associated with poor patient outcomes.^7^^,^^17^^,^^25^ Posterosuperior escape of comminuted greater tuberosity fractures is noted as a specific mode of failure.^2^^,^^24^ Suture anchor repair of these fractures remains an option and has been demonstrated to require higher loads to create fracture gapping and construct failure compared to independent cannulated screw and washer fixation.^14^ However, a biomechanical study has demonstrated that locking plate constructs are substantially stiffer than suture anchors, which may be beneficial in patients with a comminuted fractures and those with poor bone quality.^6^

Multiple theories exist that aim to explain the decreased shoulder function seen with posterosuperior greater tuberosity escape. Superior displacement has been theorized historically to lead to earlier subacromial impingement with shoulder abduction and forward flexion.^17^ Detensioning of the inserting rotator cuff musculature and alteration of the length-tension relationship may be the primary driver of poor shoulder function. In our limited study, we note that placement of the plate directly on the superior aspect of the greater tuberosity was not associated with limitations in abduction or forward flexion at final follow-up. This may be because the prevention of posterosuperior escape in these patients effectively maintains the tension of the inserting rotator cuff musculature. Furthermore, the plate utilized in this technique is low-profile (<2 mm thick), thus minimizing the risk of subacromial impingement.

Minimizing fracture displacement can be challenging in a patient with osteopenia and diminished rotator cuff tendon integrity. Biomechanically, suture anchor repair has been demonstrated to require higher loads to create fracture gapping and construct failure compared to cannulated screw and washer fixation.^3^^,^^9^^,^^14^ These studies, however, utilize osteotomized greater tuberosity fragments which are not felt to be representative of the more comminuted fracture pattern with an impacted fracture bed seen in vivo. Bahrs et al in their cohort of 141 injuries, demonstrated that 59% of greater tuberosity fractures were the result of a known anterior glenohumeral dislocation.^1^ The study additionally described that 53% of greater tuberosity fractures were composed of 3 or more fragments, and that the entire tuberosity was involved in a majority of cases. These fracture characteristics are not recreated in existing biomechanical studies and could be overstating the efficacy of suture anchor constructs.^3^^,^^9^^,^^14^ Those studies describing the outcomes following suture anchor repair tend to lack any comparison group, although moderate to good patient outcomes are reported.^4^^,^^10^^,^^12^^,^^13^ An additional biomechanical study has demonstrated that locking plate constructs have increased stiffness compared to double-row suture bridge constructs.^6^ Biomechanical and electromyographic studies suggest that the forces generated by the supraspinatus, infraspinatus, and teres minor, with certain specific shoulder motions, may exceed the tolerances of suture anchor fixation, but not a locking plate construct.^18^^,^^23^ Further biomechanical and comparative patient studies are warranted to delineate which construct is superior and under what circumstances each should be utilized.

Utilizing a locking plate, augmented with suture anchors through the rotator cuff tendon, through a mini-open deltoid-split approach for the management of split-type greater tuberosity fractures has been described previously.^5^^,^^11^^,^^22^ Schöffl et al first published this technique in 2011 using a “Bamberg” plate, or a self-adjustable titanium calcaneus plate that can be contoured and trimmed for use.^22^ Their series of 10 patients had no complications and no secondary loss of reduction, corroborating the benefits of using a rigid, yet modular construct. To the authors' knowledge, this is the first report utilizing a patellar plate.

Our study has several limitations. Isolated greater tuberosity fractures are not well described in the literature, and their true incidence is unknown, making generalizability questionable. As a result, our study sample was small. However, we do present a heterogenic population with both young and old patients, harboring both good and poor bone quality. In addition, our mean follow-up was 12.5 months, and it is likely that longer follow-up would increase the complication and revision rate, particularly as it relates to rotator cuff pathology. Finally, in order to truly capture ROM outcomes, a goniometer of video analysis software could have been utilized.

## Conclusion

This technique review describes a strategy utilizing a low-profile variable angle locking plate for fixation of comminuted tuberosity fractures. A small series of patients who underwent treatment of their injury with the technique experienced full return to preoperative function without complication. While early results are reassuring, further clinical and biomechanical studies are required to assess the efficacy of this treatment option and to compare to alternative surgical interventions.

## Disclaimers:

Funding: No funding was disclosed by the authors.

Conflicts of interest: The authors, their immediate families, and any research foundation with which they are affiliated have not received any financial payments or other benefits from any commercial entity related to the subject of this article.

## Figures and Tables

**Supplementary Figure:**
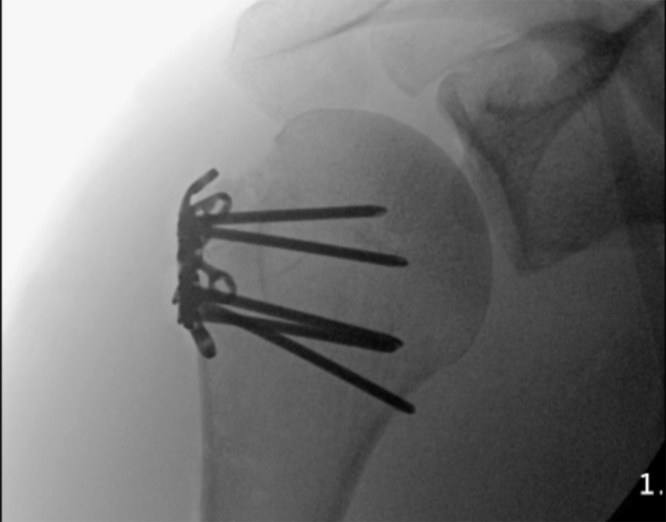


## References

[1] Bahrs C., Lingenfelter E., Fischer F., Walters E.M., Schnabel M. (2006). Mechanism of injury and morphology of the greater tuberosity fracture. J Shoulder Elbow Surg.

[2] Bono C.M., Renard R., Levine R.G., Levy A.S. (2001). Effect of displacement of fractures of the greater tuberosity on the mechanics of the shoulder. J Bone Joint Surg Br.

[3] Braunstein V., Wiedemann E., Plitz W., Muensterer O.J., Mutschler W., Hinterwimmer S. (2007). Operative treatment of greater tuberosity fractures of the humerus--a biomechanical analysis. Clin Biomech (Bristol).

[4] Carrera E.F., Matsumoto M.H., Netto N.A., Faloppa F. (2004). Fixation of greater tuberosity fractures. Arthroscopy.

[5] Chen Y.F., Zhang W., Chen Q., Wei H.F., Wang L., Zhang C.Q. (2013). AO X-shaped midfoot locking plate to treat displaced isolated greater tuberosity fractures. Orthopedics.

[6] Gaudelli C., Ménard J., Mutch J., Laflamme G.-Y., Petit Y., Rouleau D.M. (2014). Locking plate fixation provides superior fixation of humerus split type greater tuberosity fractures than tension bands and double row suture bridges. Clin Biomech.

[7] George M.S. (2007). Fractures of the greater tuberosity of the humerus. J Am Acad Orthop Surg.

[8] Huntley S.R., Lehtonen E.J., Robin J.X., Arguello A.M., Rouleau D.M., Brabston E.W. (2020). Outcomes of surgical fixation of greater tuberosity fractures: a systematic review. Orthop Traumatol Surg Res.

[9] Ishak C., Sahajpal D., Chiang A., Atallah W., Kummer F., Jazrawi L.M. (2006). Fixation of greater tuberosity fractures: a biomechanical comparison of three techniques. Bull Hosp Jt Dis.

[10] Ji J.-H., Shafi M., Song I.-S., Kim Y.-Y., McFarland E.G., Moon C.-Y. (2010). Arthroscopic fixation technique for comminuted, displaced greater tuberosity fracture. Arthroscopy.

[11] Kaisidis A., Pantos P.G., Bochlos D., Lindner H. (2018). Biomechanical analysis of the fixation strength of a novel plate for greater tuberosity fractures. Open Orthop J.

[12] Kim D.R., Noh Y.-M., Lee S.Y. (2019). Arthroscopic reduction and suture bridge fixation of a large displaced greater tuberosity fracture of the humerus. Arthrosc Tech.

[13] Kong L.-P., Yang J.-J., Wang F., Liu F.-X., Yang Y.-L. (2022). Minimally invasive open reduction of greater tuberosity fractures by a modified suture bridge procedure. World J Clin Cases.

[14] Lin C.-L., Hong C.-K., Jou I.-M., Lin C.-J., Su F.-C., Su W.-R. (2012). Suture anchor versus screw fixation for greater tuberosity fractures of the humerus--a biomechanical study. J Orthop Res.

[15] Mutch J., Laflamme G.Y., Hagemeister N., Cikes A., Rouleau D.M. (2014). A new morphological classification for greater tuberosity fractures of the proximal humerus: validation and clinical implications. Bone Joint J.

[16] Patel A.H., Lee O.C., O’Brien M.J., Savoie F.H., Sherman W.F. (2021). Short-term reoperation risk after surgical and nonsurgical management of isolated greater tuberosity fractures. JSES Int.

[17] Platzer P., Kutscha-Lissberg F., Lehr S., Vecsei V., Gaebler C. (2005). The influence of displacement on shoulder function in patients with minimally displaced fractures of the greater tuberosity. Injury.

[18] Reinold M.M., Wilk K.E., Fleisig G.S., Zheng N., Barrentine S.W., Chmielewski T. (2004). Electromyographic analysis of the rotator cuff and deltoid musculature during common shoulder external rotation exercises. J Orthop Sports Phys Ther.

[19] Robinson C.M., Shur N., Sharpe T., Ray A., Murray I.R. (2012). Injuries associated with traumatic anterior glenohumeral dislocations. J Bone Joint Surg Am.

[20] Rouleau D.M., Laflamme G.Y., Mutch J. (2016). Fractures of the greater tuberosity of the humerus: a study of associated rotator cuff injury and atrophy. Shoulder Elbow.

[21] Rouleau D.M., Mutch J., Laflamme G.-Y. (2016). Surgical treatment of displaced greater tuberosity fractures of the humerus. J Am Acad Orthop Surg.

[22] Schöffl V., Popp D., Strecker W. (2011). A simple and effective implant for displaced fractures of the greater tuberosity: the “Bamberg” plate. Arch Orthop Trauma Surg.

[23] Sharkey N.A., Marder R.A., Hanson P.B. (1994). The entire rotator cuff contributes to elevation of the arm. J Orthop Res.

[24] Verdano M.A., Aliani D., Pellegrini A., Baudi P., Pedrazzi G., Ceccarelli F. (2014). Isolated fractures of the greater tuberosity in proximal humerus: does the direction of displacement influence functional outcome? An analysis of displacement in greater tuberosity fractures. Acta Biomed.

[25] Williams G.R., Wong K.L. (2000). Two-part and three-part fractures: open reduction and internal fixation versus closed reduction and percutaneous pinning. Orthop Clin North Am.

